# Learned self-regulation in top-level managers through neurobiofeedback training improves decision making under stress

**DOI:** 10.1038/s41598-022-10142-x

**Published:** 2022-04-12

**Authors:** Pierpaolo Iodice, L. Cannito, A. Chaigneau, R. Palumbo

**Affiliations:** 1 CETAPS Lab., University of Rouen Normandy, Boulevard Siegfried, 76821 Mont Saint Aignan cedex, France; 2 Institute of Cognitive Sciences and Technologies, National Research Council, Via S. Martino della Battaglia, 44, 00185 Rome, Italy; 3 Department of Psychological Sciences, Health and Territory, University G. D’Annunzio of Chieti-Pescara, Chieti, Italy; 4 Department of Neuroscience, Imaging and Clinical Sciences, University G. D’Annunzio of Chieti-Pescara, Chieti, Italy; 5 Center for Advanced Studies and Technology (CAST), University G. D’Annunzio of Chieti-Pescara, Chieti, Italy

**Keywords:** Neuroscience, Cognitive neuroscience, Sensory processing, Stress and resilience

## Abstract

Top-level management teams are particularly exposed to stress factors as they frequently have to make important decision under stress. While an existing body of research evidence suggests that stress negatively affects decision-making processes, very little is known about possible strategies to reduce these negative effects. The aim of the current work is to investigate the effect of training self-regulation ability through neurobiofeedback on managers’ intertemporal and risky decision making. Twenty-three managers were assigned to the experimental or the control condition. All participants performed, two decisional tasks, before and after a training phase. The tasks were administered through mouse tracker software, in order to measure participants’ delay discounting and risk taking propensity on both explicit and implicit choice parameters. During the training phase, the experimental condition received a training protocol based on stress assessment tests via neurobiofeedback signals (i.e., temperature and skin conductance), with the goal of improving self-regulation ability while the control condition was administered a control training. The main result of this study is to have conclusively demonstrated that NBF training increases an individual's ability to self-regulate stress-related psychophysiological phenomena. Consequently, the improved ability to manage one's own reaction to stress enables a reduction in instinctive behavior during a probabilistic choice task.

## Introduction

The ability to make important decisions under stress is probably one of the most valuable managerial skills. Managers have to make weighty and significant financial decisions in a wide range of extremely difficult circumstances: limited time, information overload and antagonistic interpersonal interactions^1^. Scientific literature has thus been interested in investigating the processes underlying decision making under stress for more than 50 years. An important question is whether stress can push/lead decision-makers towards risk proneness or, alternatively, whether the bias previously identified in risk-taking could be exacerbated under stress^2^. Indeed, an existing body of scientific evidence accumulated over the years shows that stress influences decision making on a wide, sometimes contrasting, range of different outcomes^3–6^. Whereas a handful of studies have shown the positive effects of stress, several studies have revealed that stress negatively influences decision making^7,8^. Many studies indicate that decisions made under stress tend to be much more irrational and rely more on the instinct that led to opt for the alternative that will offer the best possible outcome as perceived by the decision maker. Previous results suggests that a person under stress could make decisions in an unsystematic and rushed manner and be led to not consider all the options^3,9,10^. Stress can lead to a number of unintended consequences—most of which will be outside decision makers' awareness—including a restriction or narrowing of attention span, increased distraction and increased reaction time^11^.

In one of the most pivotal studies in this field of research, by Janis and Mann (1977) found that simply having a coping pattern of vigilance allows for proper and rational decision-making^9^. The authors theorize that, under stress, vigilance, or an awareness, could be replaced by hypervigilance, which generates a hurried, disorganized and incomplete evaluation of information that leads to incorrect decisions^9,12^. In this context, efficient executive cognitive skills supported by adaptability to stress factors^13^ are crucial to ensure optimal cognitive and physical performance^14,15^. Efficient goal-oriented behavior and adaptability require, from a dynamic system perspective, an organism in which all subsystems work elastically and functionally with a large number of degrees-of-freedom^16^. At the core of the dynamic system is a central autonomic network (CAN), which includes the sympathetic and parasympathetic subnets whose activities create a dynamic equilibrium^17–20^. The former drives activation, while the latter actively initiates relaxation via the vagus nerve, which acts to slow down the heart rate, and plays a key role in preserving homeostasis^16,20^.

The ability to maintain, or regain, an optimal state of vigilance, attentional control and perceptual abilities, would therefore be associated with the psychophysiological state of the decision makers and their ability to self-regulate.

Psychophysiological self-regulation refers to a person's ability to regulate affective and cognitive states and to adapt to different environmental conditions, thus allowing a flexible homeostasis that preserves behavior in response to different situational needs^18,21^. As stress comes into play, the homeostatic equilibrium is altered, the mind and body react to the stress and the ability to make rational choices progressively decreases as well. Moreover, studies have shown that many managers who working crisis situations^22^ or military^23^ situations are better equipped to regulate this state than others.

Increasingly, there is mounting evidence that suggests that self-regulating abilities in stressful situations are teachable^24,25^. Studies have shown that the use of equipment capable of providing visual and acoustic feedback on both neural (neurofeedback—NFT) and physiological (biofeedback—BFT) patterns enables training to be structured to improve self-regulatory ability under stress^25^. These results have been corroborated in different studies involving elite athletes^24,26^. However, little is known about how neurobiofeedback training (NBFT) responses and awareness of self-regulatory mechanisms influence decision making behavior in probabilistic and temporal discounting tasks. Discounting refers to the devaluation of an outcome when the result is delayed (delay discounting) or obtained probabilistically (probability discounting). This tendency to discount delayed or probabilistic outcomes has been studied by examining the behavior of humans and animals when choosing between an immediate reward and a delayed reward and in other studies where subjects choose between certain and probabilistic real or hypothetical results (see Green and Myerson^27^).

Across time, different evidence of a relationship between discount rewards and stress state have been reported^3,9,10^ and different theories on how stress impacts decision-making have been tested. In a recent paper Wichary and Rieskamp suggested that stress affects probabilistic discounting through an attentional-narrowing process^28^. Similarly, Kimura and colleagues reported an influence of stress responses on delay discounting^29^. Together, these results suggest an important link between the ability to self-regulate in stressful circumstances and decision making tasks.

Since previous literature showed that NBF training (NBFT) increases interoceptive awareness through self-regulation ability’s improvement^30^ and self-regulation ability modulates psychophysiological alterations due to stress^22,31^. Previous research has sought to explain the cognitive adaption processes that underlie neurofeedback and biofeedback^32^ but just in recent times some multicomponent model has been proposed. For example, Gaume and colleagues proposed a model which, taking into account several biological processes and cognitive components, suggests that there are five key elements to be considered in NBF protocols which pertains (1) perceptibility (2) autonomy (3) mastery (4) motivation and (5) learnability^33^. To best of our knowledge there are no evidence that allows a direct comparison between mechanism involved in NBF training underlying processes and other regulation techniques, such as, for example cognitive reappraisal (e.g. Ochsner and Gross)^34^. Here, we sought to investigate whether one month of NBFT would influence participant’s behavior in decision making tasks, amongst a condition of managers in a multinational company.

Capitalizing on previous studies on temporal^35^ and probabilistic^36^ discounting tasks, using MouseTracker software^37^ we adapted the task to be able to record the x and y coordinates of the mouse’s movements trajectories associated with the participants’ choices.

The managers performed the task before following the NBFT and at the end of the training session. We expected that after training, participants would better self-regulate their internal state, reducing automatic response pattern and exhibiting a shift in discounting behavior as indicated by a change in discount rate in delay discounting and probabilistic discounting economic tasks. As a consequence, reaction times were expected to be slower (longer) than in the control condition.

## Results

### Psychological assessment

As shown in Table 1, the two conditions were not significantly different for all the considered variables.

**Table 1:** Results of independent t-test on the questionnaires’ subscales between NBF and Control conditions.

Questionnaire	Subscale	*t*	*DF*	*p*	Mean difference
MAIA	Noticing	0.785	21	0.441	0.369
	Attention regulation	1.65	21	0.113	0.668
	Emotional awareness	0.173	21	0.864	0.082
	Self-regulation	1.88	21	0.074	0.687
	Body listening	0.915	21	0.371	0.473
	Trusting	1.60	21	0.124	0.724
GDMS	Rational	− 1.51	21	0.144	− 2.26
	Intuitive	− 1.37	21	0.183	− 3.33
	Dependent	− 0.789	21	0.439	− 0.972
	Avoidant	− 1.16	21	0.258	− 1.86
	Spontaneous	− 0.609	21	0.549	− 0.623
PANAS	Positive	− 0.408	21	0.687	− 1.13
	Negative	− 1.94	21	0.065	− 3.84
BIS	Total	− 1.38	21	0.181	− 3.18

*DF* degree of freedom.

### Mouse tracker, delay and probability discounting tasks

#### Delay discounting

**Figure 1:**
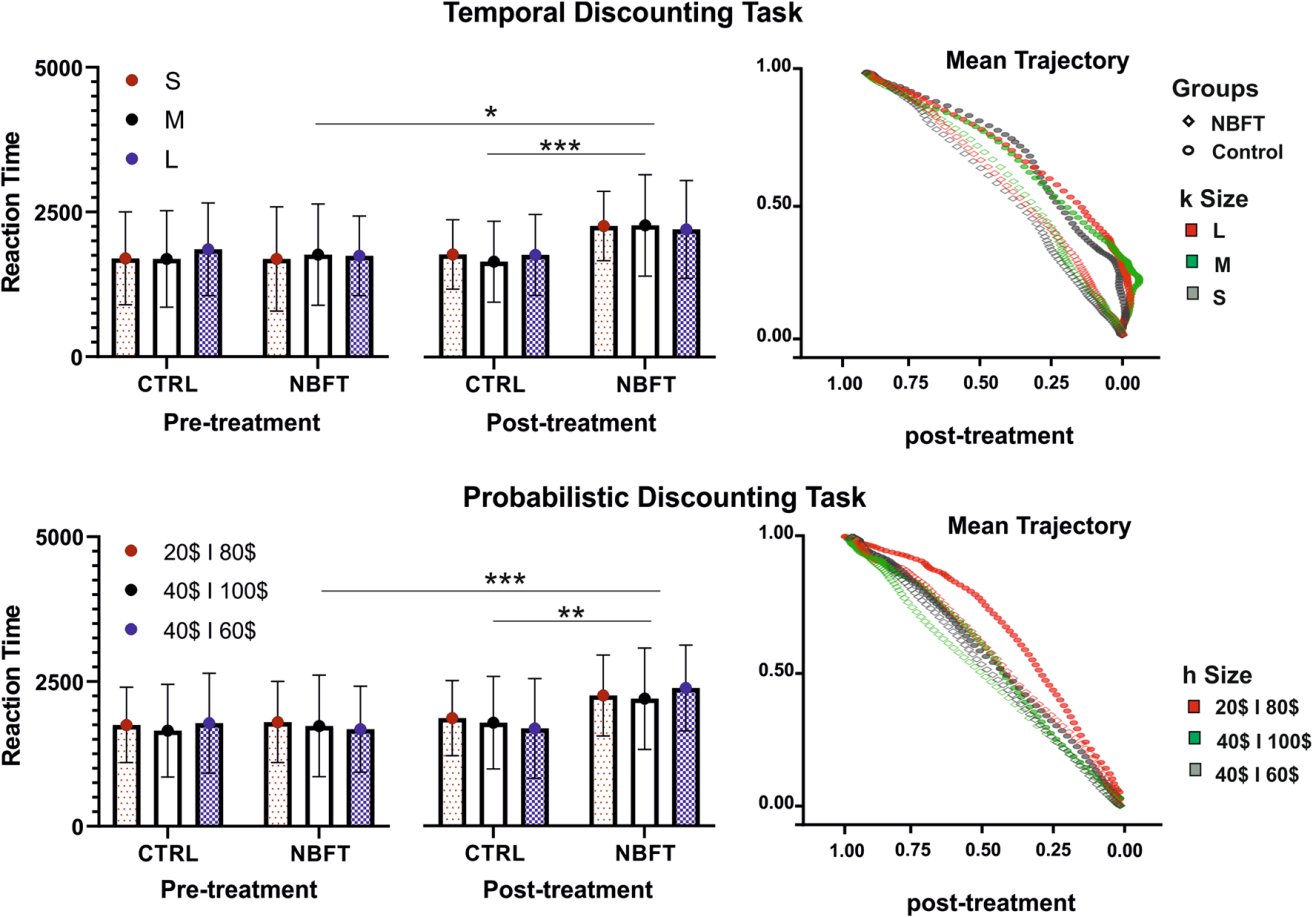
Effects of NBFT on Temporal and Probabilistic Discounting Task. Reaction time was presented in two distributions pre and post treatment. Data was reported for the three different sizes (L, M, and S for Temporal and 20€/80€, 40€/100€ and 40€/60€ for Probabilistic). The right panel shows the mean trajectory of mouse kinematic after treatment, spatial trajectories of the responses of NBFT (diamonds) vs. control (spheres) conditions. In temporal discounting tasks, in the Control but not in the NBFT condition, participants show a marked initial attraction towards the “later” choice (y-axis: 1 = Now,—1 = Later) alternative, which is eventually revised on-line. See the main text for details.

The upper panel of Fig. 1 shows mean estimates of the degree to which delayed rewards were discounted by the managers trained by NBF and control (see Table 2 for exact values). Although the condition-averaged *k*-values tended to be lower for NBF condition, this difference was not statistically significant (*p* = 0.227).

**Table 2:** Results of the repeated measures ANOVAs conducted on Discounting Rate, Reaction Time (RT), maximum deviation (MD) and the area under the curve (AUC), using the time (pre vs. post-treatment) and the condition (NBFT vs. control) as factors.

	Delay discounting			Probability discounting								
	2 × 2 ANOVA degree of delay discounting			2 × 2 ANOVA degree of probability discounting								
	F	P	p$\upeta ^{2}$	F	P	p$\upeta ^{2}$						
Group * size/part	0.723	0.487	0.011	0.563	0.572	0.014						
Group	0.421	0.518	0.003	5.836	0.019	0.073						
Part	0.282	0.755	0.004	5.197	0.008	0.129						

	2 × 2 ANOVA effect of training on reaction time						2 × 2 ANOVA effect of training on reaction time					
	After			Now			Risk			Safe		
	F_After_	P_After_	p$\upeta _{{{\text{After}}}}^{2}$	F_Now_	P_Now_	p$\upeta _{{{\text{Now}}}}^{2}$	F_Risk_	P_Risk_	p$\upeta _{{{\text{Risk}}}}^{2}$	F_Safe_	P_Safe_	p$\upeta _{{{\text{Safe}}}}^{2}$
Time * Group	11.615	0.014	0.014	6.075	0.014	0.004	9.950	0.002	0.007	11.709	< 0.001	0.009
Time	5.068	0.006	0.006	1.834	0.176	0.001	15.130	< 0.001	0.010	16.118	< 0.001	0.012
Group	2.494	0.003	0.003	9.313	0.002	0.006	8.126	0.004	0.005	3.217	0.073	0.002

	2 × 2 ANOVA effect of training on MD						2 × 2 ANOVA effect of training on MD					
	After			Now			Risk			Safe		
	F_After_	P_After_	p$\upeta _{{{\text{After}}}}^{2}$	F_Now_	P_Now_	p$\upeta _{{{\text{Now}}}}^{2}$	F_Risk_	P_Risk_	p$\upeta _{{{\text{Risk}}}}^{2}$	F_Safe_	P_Safe_	p$\upeta _{{{\text{Safe}}}}^{2}$
Time * Group	0.133	0.716	0.005	3.916	0.048	0.002	4.720	0.030	0.004	5.367	0.02	0.004
Time	3.833	0.051	0.007	5.239	0.022	0.003	0.023	0.880	< 0.001	5.168	0.023	0.04
Group	5.792	0.016	< 0.001	63.342	< 0.001	0.037	5.354	0.021	0.004	4.148	0.042	0.003

	2 × 2 ANOVA effect of training on AUC						2 × 2 ANOVA effect of training on AUC					
	After			Now			Risk			Safe		
	F_After_	P_After_	p$\upeta _{{{\text{After}}}}^{2}$	F_Now_	P_Now_	p$\upeta _{{{\text{Now}}}}^{2}$	F_Risk_	P_Risk_	p$\upeta _{{{\text{Risk}}}}^{2}$	F_Safe_	P_Safe_	p$\upeta _{{{\text{Safe}}}}^{2}$
Time * Group	< 0.001	0.981	< 0.001	6.516	0.011	0.004	17.978	< 0.001	0.012	25.766	< 0.001	0.019
Time	6.956	0.009	0.008	4.860	0.028	0.003	0.385	0.535	0.002	2.907	< 0.001	0.002
Group	1.871	0.172	0.002	24.237	< 0.001	0.015	33.411	< 0.001	0.022	3.710	0.054	0.003

The results are reported for the temporal (A) and probabilistic task (B).

No significant main effect of delayed reward size on degree of delay discounting was observed (*F*_(2,822)_ = 0.132, n.s.) No significant condition by reward-amount interaction was detected (*p* = 0.87). Across conditions and delayed reward amounts, *k*-values were not significantly correlated with NBF results.

The 2 × 2 ANOVA conducted by RT to compare the effect of NBF training across the conditions revealed a statistically significant condition by time interaction (*F*_1,830_ = 11.615, *p* = 0.001, *pη*^2^. = 0.014). The main effect of time (*F*_1, 830_ = 5.068, *p* = 0.05, *pη*^2^. = 0.006), indicating that the RT associated with the choices after training were significantly longer compared to the presession. The Bonferroni post-hoc tests indicated that the RT was longer in the NBF condition after training (*t*_19_ = 3.99, *p* < 0.001), while no change was observed for the CTRL condition (*t*_19_ = 0.845, n.s.). No statistical differences were found in the kinematic data (MD and AUC) in the two conditions before and after the experimental period.

Additionally, to ensure that the results discussed above were not influenced by the study design (i.e., carryover effect) a further analysis was conducted on the discounting parameters variables.

Delay discounting: The results of the 2 × 2 ANOVA with condition (NBFT vs. CTRL) and time order (“first” vs. “second”—counterbalanced) show no statistically significant main effect of time order (RT, F_1, 63_ = 0.77, n.s.; MD, F_1, 63_ = 0.32, n.s.; AUC: F_1, 63_ = 0.89, n.s.) and condition (RT, F_1, 63_ = 1.02, n.s.; MD, F_1, 63_ = 0.44, n.s.; AUC: F_1, 63_ = 0.81, n.s.) nor interaction with time order (RT, F_1, 830_ = 1.02, n.s.; MD, F_1, 830_ = 0.44, n.s.; AUC: F_1, 830_ = 0.81, n.s.).

Probability discounting: The results of the 2 × 2 ANOVA with condition (NBFT vs. CTRL) and time order (“first” vs. “second”—counterbalanced) replicated the previously discussed main effects of condition (RTs: F_1, 63_ = 7.77, *p* < 0.01; AUC: F_1, 63_ = 31.89, *p* < 0.001). The results show no statistically significant main effect of time order (RT, F_1, 63_ = 1.24, n.s.; MD, F_1, 63_ = 0.81, n.s.; AUC: F_1, 63_ = 1.09, n.s.) and nor interaction with time order (RT, F_1, 830_ = 1.02, n.s.; MD, F_1, 830_ = 0.44, n.s.; AUC: F_1, 830_ = 0.81, n.s.).

This result indicated that the conditions time order in experimental design (for both NBFT and control condition) did not have any significant influence on discounting parameters.

#### Probability discounting

The lower panel of Fig. 1 illustrates the significant differences in degree of probability discounting across the NBF condition and matched controls after training period (*F*_(1,63)_ = 5.836, *p* < 0.02, *pη*^2^ = 0.073). Managers trained with NBF discounted the hypothetical probabilistic monetary rewards more steeply than controls. The main effect indicated that, consistent with our hypothesis, the *h*-values average associated with conditions were significantly higher in the NFB Condition compared to the control condition, indicating a reduction in automatic choice behavior in the NFB condition after the training period. A significant main effect of the three sizes in the probability discounting task (*F*_(1,63)_ = 5.197, *p* < 0.01, *pη*^2^ = 0.129) was detected but the condition × size interaction was not significant (*p* = 0.572).

The effect of NBF training on managers' behavior is shown in Fig. 1. The figure shows the increase in the number of responses given by managers that have associated a higher expected value. Show that they opted to behave in a way that was less instinctive.

The effect of the training on reaction times of the probabilistic task was revealed by a significant interaction (Condition*Time) for both risk (*F*_(1, 1484)_ = 9.950, *p* = 0.002, *pη*^2^. = 0.07) and safe (*F*_(1, 1328)_ = 11.709, *p* = 0.001, *pη*^2^. = 0.009) responses. For risk responses, the main effect of the training was significant (*F*_(1, 1484)_ = 8.126, *p* = 0.004, *pη*^2^. = 0.005), and there was a significant main effect on time (*F*_(1,1484)_ = 15.130, *p* < 0.001, *pη*^2^. = 0.01). The Bonferroni post-hoc tests indicated that the RT was longer in the NBF condition after training (*t*_19_ = 4.883, *p* < 0.001), while no change was observed in the CTRL condition (*t*_19_ = 0.531, n.s.).

For safe responses, the main effect of time was significant (*F*_(1, 1328)_ = 16.118, *p* < 0.001, *pη*^2^. = 0.012), whereas there was no significant main effect of the condition (*F*_(1, 1328)_ = 3.217, *p* = n.s., *pη*^2^. = 0.002). The Bonferroni post-hoc tests indicated that the RT was longer in the NBF condition after training (*t*_19_ = 5.523, *p* < 0.001), while no change was observed in the CTRL condition (*t*_19_ = 0.401, n.s.).

The effect of the training on kinematic analysis of the probabilistic task shows a significant interaction (Condition*Time) for both MD (*F*_(1, 1328)_ = 5.367, *p* = 0.02, *pη*^2^. = 0.021) and AUC (*F*_(1,1328)_ = 25.766, *p* = 0.001, *pη*^2^. = 0.019) responses. For MD, the main effect of condition was significant (*F*_(1,1328)_ = 4.148, *p* = 0.05, *pη*^2^. = 0.042), and there was a significant main effect on time (*F*_(1,1328)_ = 5.168, *p* = 0.023, *pη*^2^. = 0.023). The Bonferroni post-hoc tests indicated that the RT was longer in the NBF condition after training (*t*_19_ = 3.409, *p* < 0.01), while no change was observed in CTRL condition (*t*_19_ = 0.020, n.s.).

For AUC, the main effect of the condition was significant (*F*_(1, 1328)_ = 3.710, *p* = 0.05, *pη*^2^. = 0.03), whereas there was no significant main effect of time (*F*_(1,1328)_ = 2.907, *p* = n.s., *pη*^2^. = 0.002). The Bonferroni post-hoc tests indicated that the RT was longer in the NBF condition after training (*t*_19_ = 5.036, *p* < 0.001), while no change was observed in CTRL condition (*t*_19_ = 0.137, n.s.).

### Neurobiofeedback training

For all subjects the response to the stressful stimulus of the observed parameters was in conformity with some of the response documented in the literature (by direction and amplitude): for example decrease in temperature as previously hypothesized in another study^25^; increase in skin conductance (e.g., Raaijmaker et al.)^38^ and EEG signals (e.g., Sherlin, Muench and Wyckoff, 2010)^39^. For more details, a recent systematic review is available Kennedy 2019^40^.

**Figure 2:**
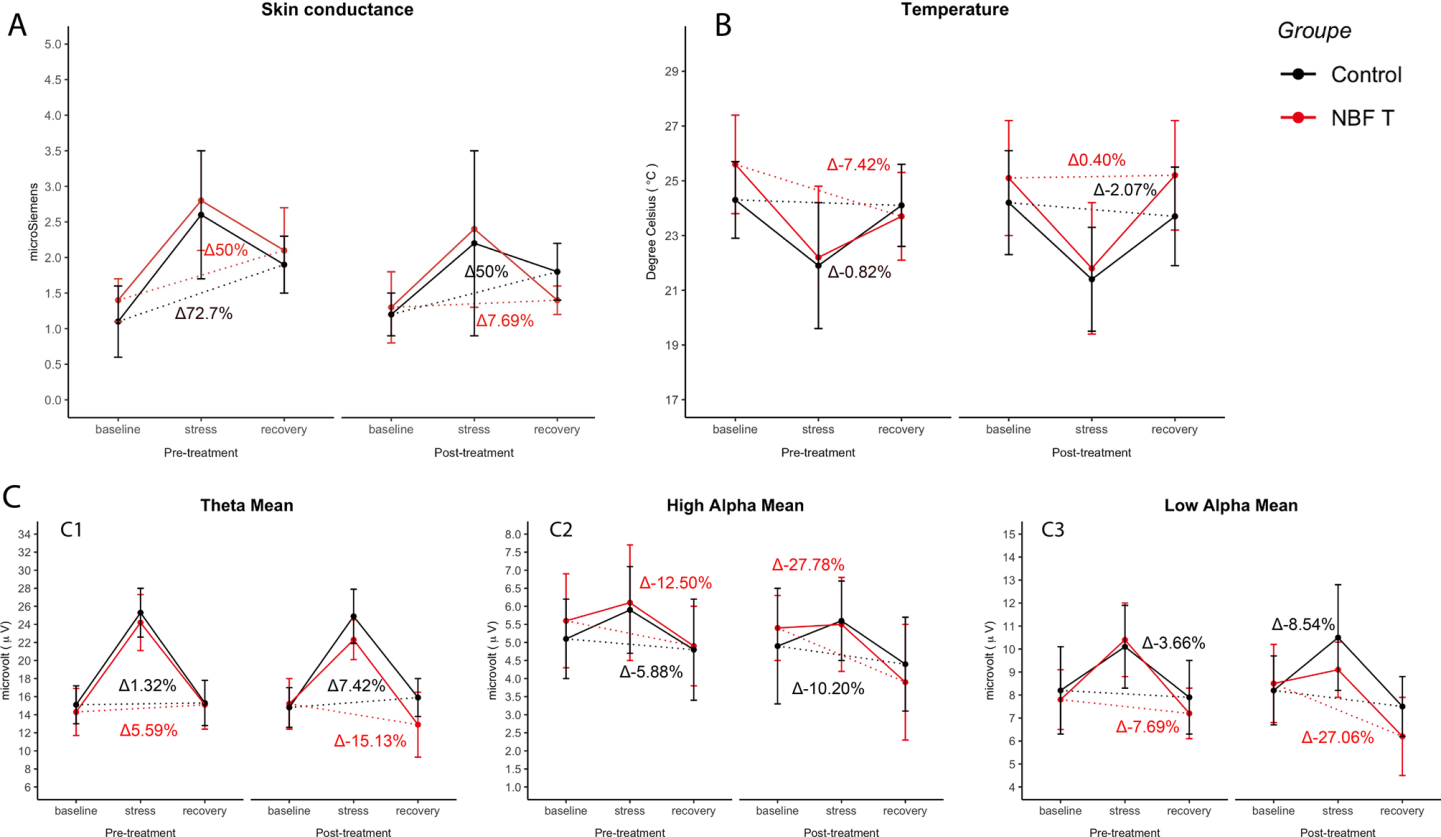
Effects of treatment (red dots, NBFT; black dots, control condition). (**A**,**B**,**C**) neurophysiological profile: SCL (**A**), temperature (**B**), and EEG (**C**)— Theta (**C1**), High Alpha (**C2**) and Low Alpha (**C3**)—during baseline, stress, and recovery phase, which were measured before (Pre) and after treatment (post). Δ denotes the differences between recovery and baseline; smaller differences indicate more efficient self-regulation. Vertical bars measure standard error. *Significant differences.

Repeated measures ANOVA 2 (Conditions: NBFT vs. Control) × 2 (test session: pre-treatment vs. post treatment) × 3 (conditions: baseline, stress and recovery; within-subjects) was performed to assess the effect of treatment on their self-regulatory ability (Fig. 2).

First, we observed the effect of the stressor task (Stroop) on Skin Conductance Level (SLC), Temperature and EEG signals. We found a significant interaction between conditions and test sessions (*F*_(1,160)_ = 5.511, *p* < 0.05 *pη*^2^ = 0.12). We found a main effect of conditions for SCL (*F*_(1, 160)_ = 101.587, *p* < 0.01, *pη*^2^ = 0.10), for temperature (*F*_(1, 160)_ = 85.299, *p* < 0.01, *pη*^2^ = 0.07), for theta mean (*F*_(1, 160)_ = 114.669, *p* < 0.01, *pη*^2^ = 0.12), for high alpha mean (*F*_(1, 160)_ = 11.841, *p* < 0.05, *pη*^2^ = 0.03) and for low alpha mean (*F*_(1, 160)_ = 99.541, *p* < 0.01, *pη*^2^ = 0.09). Post hoc analysis showed a significant lower stress effect in post-treatment in SCL (*t*_19_ = 5.423, *p* < 0.01), theta mean (*t*_19_ = 3.284, *p* < 0.05) and low alpha mean (*t*_19_ = 3.114, *p* < 0.05).

Second, the ANOVA revealed an interaction between condition and session (*F*_(1, 160)_ = 6.188, *p* < 0.05, *pη*^2^ = 0.03). The planned contrasts used to assess the specific effects of treatment found that the two conditions diverged in their difference between baseline and recovery in the post-treatment session for all observed parameters: SCL (*F*_(1, 68)_ = 76.229, *p* < 0.01, *pη*^2^ = 0.13); temperature (*F*_(1, 68)_ = 8.744, *p* < 0.05, *pη*^2^ = 0.02); theta mean (*F*_(1, 160)_ = 12.461, *p* < 0.05, *pη*^2^ = 0.03); high alpha mean (*F*_(1, 160)_ = 45.273, *p* < 0.05, *pη*^2^ = 0.08) and for low alpha mean (*F*_(1, 160)_ = 61.155, *p* < 0.05, *pη*^2^ = 0.06). The same contrast in pre-treatment session showed no significant difference between conditions.

Additionally, in order to ensure that the results discussed above were not influenced by the study design (i.e., carryover effect), a further analysis was conducted on the neurophysiological variables.

The results of the 2 × 2 × 2 ANOVA with condition (NBFT vs. CTRL) and time order (“first” vs. “second”—counterbalanced) and conditions (baseline vs. stress) show no statistically significant main effect of time order (SCL, F_1, 830_ = 0.03, n.s.; Temperature, F_1, 830_ = 0.22, n.s.; EEG, F_1, 830_ = 0.69, n.s.; Theta, F_1, 830_ = 0.14, n.s.; High Alpha, F_1, 830_ = 1.11, n.s.; Low Alpha, F_1, 830_ = 0.91, n.s.) and conditions (SCL, F_1, 830_ = 0.71, n.s.; Temperature, F_1, 830_ = 0.29, n.s.; EEG, F_1, 830_ = 0.84, n.s.; Theta, F_1, 830_ = 0.02, n.s.; High Alpha, F_1, 830_ = 0.58, n.s.; Low Alpha, F_1, 830_ = 0.31, n.s.) nor interaction with time order (SCL, F_1, 830_ = 0.74, n.s.; Temperature, F_1, 830_ = 0.96, n.s.; EEG, F_1, 830_ = 0.27, n.s.; Theta, F_1, 830_ = 0.47, n.s.; High Alpha, F_1, 830_ = 0.61, n.s.; Low Alpha, F_1, 830_ = 0.66, n.s.).

This result indicated that conditions’ time order as based on the experimental design did not have any significant influence on neurophysiological profile in our study.

## Discussion

In the present study, we explored for the first time whether teaching/practising self-regulation ability through NBF may influence temporal and probabilistic economic decision making. We demonstrate that providing NBF training in high level managers (1) increased self-regulation and adaptability during cognitive stress conditions and (2) decreased subjective discounting rating in probabilistic choices with a lower number of impulsive responses. These findings provide compelling evidence for the influence of the ability to self-regulate on decision making in economic choices scenarios and contribute to the understanding of the relationship between stress, psychophysiological reactions, and economic decision making^1^. In addition, we believe that our approach introduces a new procedure for training high level managers to protect and optimize their perceptual analysis (vigilance) during stressful conditions.

### Neurobiofeedback training

Our results corroborate results from previous studies, suggesting that when humans have the possibility to receive interoceptive and neural feedback of their neurovisceral status during targeted training, this allows adaptation of the control mechanisms of self-regulation (e.g., Mirifar et al.)^41^. The theoretical framework was based on the neurovisceral integration model that integrates extensive evidence linking the autonomic and central nervous systems into a functional and structural network involved in the emotional regulation of behavior^18,19,42,43^.

All physiological parameters (temperature, skin conductance and EEG) recorded during training show how the managers trained with NBF reduced the activation time of self-regulating mechanisms in stressful conditions. In absolute terms, they also demonstrated a greater capacity compared to the extent of possible adaptations.

Our data are in line with Janka and colleagues’ results in crisis management, which suggest that the increased resistance to stress perception is because of the ability to adapt to a crisis environment^31^. We suggest that use of NBF in cognitive stress conditions leads to adaptations in the perception of the participants' neurovisceral states and the managers’ ability to self-regulate these states by increasing the managers’ resilience in stressful situations. In keeping with this, it has shown that NFT’s effectiveness in improving symptoms in clinical samples and in enhancing performance in non-clinical samples, for example in musicians^44^ and athletes^41^. Finally, recent research demonstrates that NFT/BFT (then NBFT) reduces anxiety, improves attention, and ultimately enhances performance skills (for reviews, see Morgan and Mora^45^; Mirifar et al.^41^). Together with our results, these findings suggest that improved self-regulation, neuro signals and interoceptive signals, may help to improve cognitive perception in stressful situations.

### Economic decision making

Having the ability to self-regulate influences behavior during a probability discounting task. In particular, we found that individuals trained in NBF discounted hypothetical probabilistic monetary rewards significantly compared to the matched control participants. This result is confirmed by the increase in the number of responses given associated with an optimal expected value, which is in line with previous findings of an association between neurovisceral awareness and stress perceptions^31^. Despite the heterogeneous models of the stress genesis, there is a broad consensus that stress elicits psychological, physiological and behavioral reactions^46^. Recent reviews and meta-analyses postulate that stress occurs whenever a demand exceeds the regulatory capacity of an organism^47,48^.

We suggest that increasing the ability to regulate stress-related physiological phenomena allows the individual to reduce the level of perceived stress through the reduction of psychophysiological responses to the stressful stimulus.

Numerous studies conducted on decision-making in high-risk situations report that stress enhances risk-taking for rewards that result in disadvantageous performance on the task compared to controls^49^. In the same vein, previous research indicates that in decision-making under uncertainty, stress enhances risk-taking for high potential reward options in males^8,50^.

The results of our study show how managers, after being trained with NBF, are able to counteract this trend. In fact, they show more *rational* behavior compared to the control condition. This trend is confirmed by the analysis of the kinematic MouseTrackers data: the NBF condition has longer reaction times (similar to pre-treatment compared to the control condition, which seems to make more instinctive responses). Despite longer reaction times the NBF condition has significantly shorter MD values than the control condition. This data has been pinpointed with greater confidence in the choice made.

Like previous studies reporting on delay discounting literature (e.g., Kimura et al., 2013), both conditions discount delayed monetary rewards significantly. On delay discounting tasks, acute stress had been shown to lead to greater discounting rates^29^. Following an acute stressor, individuals prefer small, immediate rewards (i.e., receive €2 now) over large, delayed rewards (i.e., receive €10 after 30 days) compared to controls. Takahashi suggested several possible mechanisms that could modulate delay discounting^51^. The most prominent mechanism is the altered neurotransmission of dopaminergic neurons in the neural circuit responsible for reward processing. Psychological stress enhances cortisol secretion and via transient elevation of dopaminergic activity could increase the level of impulsivity in an intertemporal choice task^52^. More recently, Haushofer and colleagues showed that induction of stress leads to increase preferences for smaller sooner rewards while not affecting delay discounting itself, thus suggesting that the influence of stress level on discounting choices may not be related to present bias solely^53^. Furtherly, it should be noted that other evidence suggested no impact of stress (in the form threat of unpredictable shock) on temporal discounting, suggesting that a certain level of stress may perturbed low-level processing while not impacting on high level executive functioning^54^.

In our study, we do not provide evidence of the effects of NBF training on temporal discounting. No statistical differences were found in the k-values of the two study conditions before and after the experimental protocol. This may suggest that the self-regulatory mechanisms used by the managers are not sufficient to significantly counteract the hormonal response due to stress.

However, it is important to report a significant increase in response time in subjects trained with NBF firstly. This suggests more deliberative behavior amongst the participants. Future studies with a larger population may help to clarify these results.

Taken together, the results of this study uniquely demonstrate that NBF training increases the individuals’ ability to self-regulate stress-related psychophysiological phenomena. As a result, the improved capacity to manage one’s reaction to stress makes it possible to reduce stress-related behavioral patterns during an economic decision making task under risk. Managers succeed in preserving a rational and less instinctive decision making strategy in the probabilistic task.

## Materials and methods

### Participants

Twenty-three male participants were recruited from a multinational company with five different locations around the world. Participants were top-level managers working at the company’s head office in Italy. All participants were volunteers and provided informed consent before taking part in the protocol. They were in good health, Italian speakers, right-handed with normal or corrected-to-normal vision. Although they held different positions, all the participants were comparable overall due to the years of experience working in the company (M = 7 ± 3.2 SD). None of participants reported previous expertise about or knowledge of meditation, yoga and mindfulness practices and no one reported any vision or hearing loss due to illness or disease.

Because previous research on decision making lacks information about the power-effect size of their analyses, we have opted to use previous studies using similar research designs on the effect of NBFT (see Christie et al*.*^24^). A priori power analysis for repeated-measures analysis of variance was performed for sample size calculation [G*Power 3.1.9.2 (www.gpower.hlu.de/en.html)], based on a desired pη^2^ of 0.14 (corresponding to a Cohen's f = 0.40, and to a Power (1-β err. Prob.) = 0.95) for the F-tests, and on a desired r^2^ of 0.25 for the correlation analysis, which are interpreted as an index of a large effect size^55,56^.

**Figure 3:**
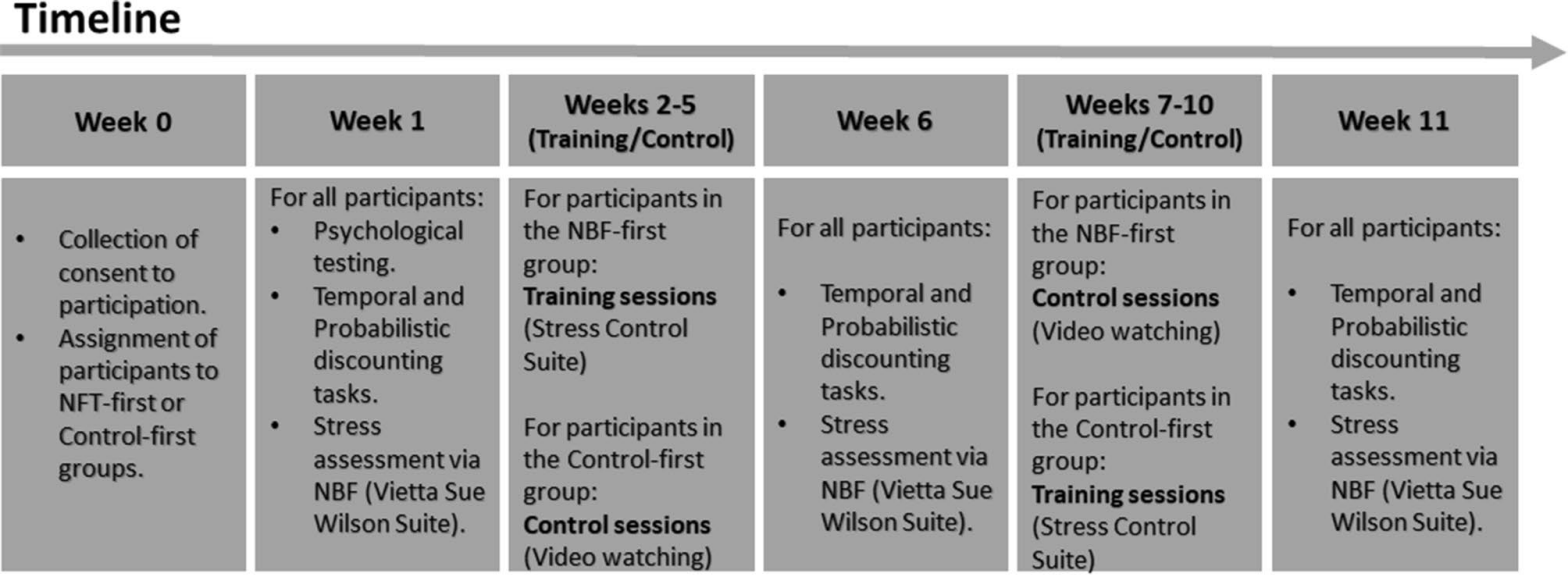
Timeline of the experimental procedure.

The results of these analyses indicated a required sample size ranging from 16 to 22 participants; we also observed 20 to be a critical number of participants, as both the effect size and observed power remain stable above this number. In view of the specific criteria required of the condition (holding a top-level management position) and the impossibility of reaching this critical threshold of 20 subjects per condition, we designed a cross study in which two conditions of 12 top-level managers initially acted as an experiment condition and control condition and then reversed roles after a two-week rest period. Participant’s assignment to the two conditions was counterbalanced (see Fig. 3 for detailed timeline) and, in addition, a statistical testing was conducted to exclude time effects before cumulated data were used for statistical analysis (see “Results” section).

Informed consent was obtained from each participant and the study protocol conforms to the ethical guidelines of the Declaration of Helsinki as reflected in its approval by the human research committee of the Institutional Review Board of Psychology (IRBP), University G. d'Annunzio of Chieti-Pescara (Protocol n° 20019—CRRP@unich.it).

### Apparatus and stimuli

All the behavioral tasks and physiological recordings were conducted in the same room located at the company headquarters. Throughout the months long experiment, the conditions in the room were stable. The temperature was set to 20 °C and natural light penetration was prevented and all the sessions were conducted with the same artificial light to avoid possible influences due to seasonal or time changes both on the participants’ mood as well as on the quality of the physiological recordings. The chosen experimental room was never used by the participants, before or during the experiment, as a personal office or meeting room, in order to avoid a bias or influence due to familiarity with the setting. To a feasible extent, every physiological recording was scheduled on an empty stomach.

### Psychological assessment

A psychological assessment was conducted on each participant in order to ensure that those in the experimental condition did not significantly differ from participants in the control condition for what concerns various psychological features or traits which may exert their influence on choice (i.e. decision style, impulsiveness, affective state) or on neurobiofeedback training itself (i.e., interoceptive awareness). The assessment was performed in week 1 for all participants and included four psychometric questionnaires.

The Italian version of the General Decision-Making Style (GDMS^57^) questionnaire was used to track how individuals approach decision making situations. It is a self-report questionnaire composed of 25 items using the Likert scale of options (from 1 = strongly disagree to 5 = strongly agree) and 5 sub-scales (Rational, Avoidant, Dependent, Intuitive, Spontaneous style)^58^.

The Italian version of the Barratt Impulsiveness Scale (BIS-11^59^) was used to assess the construct of impulsiveness. The BIS-11 is a 30-item self-report questionnaire. All items are measured on a 4-point scale (1 Rarely/Never; 2 Occasionally; 3 Often; 4 Almost Always/Always). The total score is obtained by adding all the points from each item indicating an impulsiveness level so that the higher the total score on the BIS-11, the higher the impulsiveness level.

The Italian version of the Positive and Negative Affect Schedule (PANAS^60^), was used to evaluate participants’ affective state. The Positive Affect scale reflects the extent to which a person feels enthusiastic, excited, active, and determined while the Negative Affect scale reflects a broad range of aversive affects, including fear, nervousness, guilt and shame. It is a self-report measure composed of 20 items (10 items for positive and 10 items for the negative affective state) each rated using a 5-point Likert scale (from 1 = slightly or not at all to 5 = extremely). Finally, the Italian version of the Multidimensional Assessment of Interoceptive Awareness (MAIA^61^), Scale was used to evaluate the participant’s ability to perceive their own inner physiological state. It is composed of 32 items on a 6-point Likert scale, in which the participant had to rate “how often each statement applies to you generally in daily life,” with ordinal responses coded from 0 (*never*) to 5 (*always*). This multidimensional instrument measures the interoceptive awareness on eight scales: (1) Noticing, (2) Not-distracting, (3) Not-worrying, (4) Attention regulation, (5) Emotional awareness, (6) Self-regulation, (7) Body listening and (8) Trusting. The score for each scale is calculated by averaging the scores of its individual items, and thus can vary in the 0–5 range.

### Temporal and probabilistic discounting tasks

The MouseTracker system was used for two different stimuli: *temporal discounting and probabilistic discounting.* In both tests, the following procedure was applied to obtain the variables: number of choices, response time, and kinematics data.

**Figure 4:**
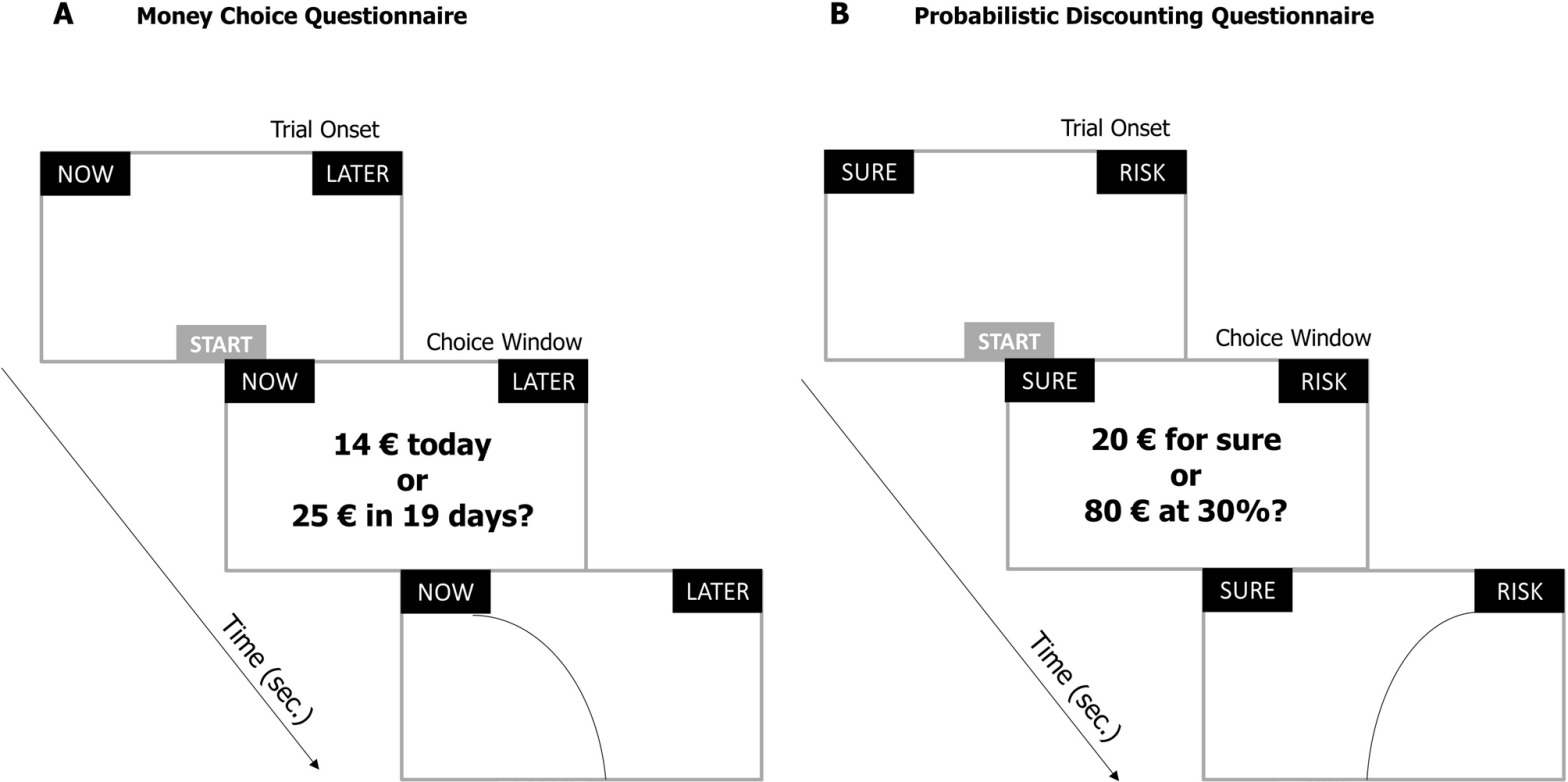
Behavioral Paradigm. (**A**) Money Choice Questionnaire: in order to correctly record the mouse’s position. At the beginning of each trial participants were instructed to press the “START” button positioned at the bottom centre of the screen to see the options. Participants were next instructed to express their preference between a smaller, but immediately available, option and a larger, but delayed alternative by clicking on the corresponding response button. (**B**) Probabilistic Discounting Questionnaire as in MCQ participants were first instructed to press the “START” button to visualize the options. In this task, however, participants were asked to choose between a smaller but certain reward and a larger but uncertain reward.

To begin each trial, participants clicked on the /START/ button at the bottom-centre of the screen. Then, an economic proposal appeared on the screen. The participants task was to move the mouse and click on the response button at the top-left or top-right corner of the screen (Fig. 4). Rightward and leftward responses were counterbalanced. Using the MouseTracker software, we recorded the x and y coordinates of the mouse trajectories associated with the participants’ choices^37^. Because each choice has a different length (as a result of different reaction times) and, as a consequence, a different number of x–y coordinate pairs, we conducted a normalized time analysis. To permit averaging and comparison across multiple trials with different numbers of coordinate pairs, trajectories were resampled to a given number of time steps (n = 101) using a linear interpolation. The normalized trajectories were then used to compute two parameters: the Maximum Deviation (MD) and the Area Under the Curve (AUC). After calculating the idealized response trajectory (a straight line between each trajectory’s start and endpoints), the MD was calculated as the largest perpendicular deviation between the actual trajectory and its idealized trajectory out of all time steps. The AUC was instead calculated as the geometric area between the actual trajectory and the idealized trajectory. Both of these measures provide an index on spatial attraction toward the competing unselected option, i.e. higher values of MD and AUC indicates higher attraction by the unselected alternative during the trajectory toward the selected choice option. However, MD and AUC are not equivalent measures, since the MD is considered an index of the maximum spatial attraction, and it may be limited to fewer time steps, while the AUC is considered an index of the overall attraction. Nevertheless, previous studies^62–64^ have shown that using the MD versus the AUC for the same data does not substantially change the results.

Stimuli of temporal discounting. Decision making tasks and participants’ temporal discounting behavior were assessed by using the and commonly used 27-item Monetary Choice Questionnaire (MCQ^65^). In the MCQ, on each item, participants chose between immediate, smaller rewards (e.g., €25 today) and delayed, larger rewards (e.g., €35 in 25 days) of three differing sizes (9 small, 9 medium and 9 large rewards). Participants were instructed to press a start button at the bottom centre of the screen to see the options and then they were asked to choose between one button labelled “Now” and another labelled “Later” positioned equidistant from the start button, at the top right and top left corners of the screen (Fig. 4, panel A).

Stimuli of probabilistic discounting. Probabilistic discounting behaviors were assessed by using the validated and widely used 30-item Probability Discounting Questionnaire (PDQ^36^). For each item in the PDQ participants were asked to choose between a guaranteed, smaller reward (e.g. € 20 guaranteed) and a larger amount of money delivered based on a probability (e.g. 10% chance of winning € 80). Probabilistic discount was used to investigate the effect of reward probability on decision making by determining the guaranteed amount to be received that is preferred to a riskier outcome. The task included 30 items and for each one, participants had to choose between a smaller amount which was guaranteed money or, “for sure” (Fig. 4, panel B).

Both behavioral tasks were presented via computer using a 15.5’’ LCD monitor (1366 × 768 pixels). Participants were seated facing the monitor, about 60 cm away. Data from the two decisional tasks were collected using MouseTracker^37^.

### Neurobiofeedback assessment

Assessment sessions, each lasting about 60 min, were conducted at week 1 (baseline pre-treatment), week 6 (post-control or post-treatment according to the condition) and week 11 (follow-up) for all participants. In those sessions participants' psychophysiological state was assessed and monitored using the "Vietta Sue Wilson Suite" of the Thought Technology System^66^ and using a set of psychological questionnaires investigating cognitive and personality traits considered relevant in the stress management process. Moreover, in those sessions participants were asked to complete two decision making tasks in order to estimate their temporal discounting and risk-taking behavior.

The “Vietta Sue Wilson Suite” was the first activity. The suite, lasts 19 min, allows one to record multiple physiological signals during 14 different activities: two initial baselines (eyes closed baseline and eyes open baseline), 7 cognitive tasks (a Stroop test, a math subtraction test, a react track game, a imagery task, a dual tracking game, a stressor anticipation task and a brief stressor exposure) and 5 recovery sessions between cognitive tasks. The Stroop color and word test (SCWT) is a psychological test widely used to assess the participant’s ability to inhibit cognitive interference that occurs when the processing of a specific stimulus feature impedes the simultaneous processing of a second stimulus attribute. Previous literature has largely confirmed its employment as stress-induction procedure (e.g. Hamid et al.)^67^. As math subtraction test was used the serial sevens test which ask participant, beginning with the number 100, to progressively subtract 7 units, and, by increasing mental effort, has been previously employed as stress inducer activity (e.g. Kennedy and Scholey)^68^. The react track game and the dual tracking game are both taken from sport psychology literature, and both require that the participants move a ball to a specific screen place while temporal pressure increases^69^. During the imagery task participants are required to think about and imagine something considered highly relevant for them and this task is included as reference to measure arousal activation^69^. At last, during stressor anticipation block participants are asked to be in alert and advised that a stressor is about to arrive. This block is used to register anticipatory response to stress. The real stressor (sudden acute noise) is indeed presented during the last block (brief stressor exposure).

The recorded signals included muscle activity (EMG electrode positioned on the left trapezius), breath parameters (rate and amplitude), cardiovascular parameters (blood volume pressure and heart rate), skin conductance and peripheral temperature parameters. The last two parameters have also been the subject of training sessions explained in further detail.

### Procedure

The entire experimental protocol was carried out at the aforementioned multinational company's site. The company provided an air-conditioned, noise-free separate room in order to meet the environmental requirements of our study.

The study design included two evaluation procedures (pre and post training), each of these sessions was divided into three separate sections with one day of rest in between. In the first section, all participants went through a psychological assessment, in the second section, included the NBF assessment in both the rest and stress conditions, and in the third section, the participants did the probabilistic and temporal discounting tasks under stress.

*Training condition.* The treatment sessions were administered via another Thought Technology System’s Suite, named “Stress Control Suite'' that helps to monitor stimulation levels and to teach self-regulation using learning strategies to reduce tension and stress. Throughout the treatment weeks (week 2–5 for the first condition and week 7–10 for the second condition) participants took part in eight treatment sessions, each lasting about 25 min. The chosen treatment protocol was divided into two steps: a training phase and a relaxation phase. The first step was composed of five biofeedback activities (called training skin conductance, training temperature, training skin conductance with an animation, training temperature with an animation, training skin conductance and temperature together with an animation) during which, using the feedback shown on the display, participants were instructed to decrease the skin conductance or increase the temperature alternatively; this first step lasted 15 min (5 activities that lasted 3 min each). The second step was designed to be an unwinding, relaxing activity to be administered at the end of each training session. For this final step a rotation between four different commonly used relaxation techniques were used: relaxation with binaural beats, relaxation with paced breathing, guided sensory relaxation and guided meditation for emotional relaxation. Each one of these relaxation protocols lasted about 10 min. As for the assessment sessions, physiological signals, specifically skin conductance and the peripheral temperature parameters, were recorded during the entire treatment session.

*Control condition.* Participants were asked to sit and watch eight different videos (2 for weeks) concerning divulgatory topics that were unrelated to stress, relaxation practices or biofeedback’s aim (e.g. videos about pollution or desertification). Each video lasted about 25 min in order to ensure temporal comparability between treatment and control sessions.

At the end of every video, participants were asked to complete a brief questionnaire about the video’s content aimed at assessing that their focus and attention were devoted to the video.

### Data processing

#### Temporal and probabilistic discounting analysis

Based on the participants’ observed behavior, we calculated k scores and h scores by using an R syntax^70^. The syntax is based on the following well-known equations, each containing a single free parameter which is interpreted as a degree of delay (k) or probability (h) discounting. When the free-parameter value increases, the subjective value of the delayed or probabilistic outcome is more steeply discounted. For the MCQ, discounting rates for each level were calculated using Mazur’s^71^ and Kirby and colleagues’^65^ hyperbolic discounting equation:

1 $$ V \, = \, A \, / \, \left( {1 \, + \, kD} \right), $$

where *V* is the present value of the delayed reward, *A* is the amount of the delayed reward, *D* is the delay, and k is the individual discounting rate^72^. The discounting rate (k) represents the slope of the hyperbolic function, the individual’s value of delayed rewards, with larger k values reflecting larger delay discounting. Therefore, k describes the steepness of the discounting curve or, in other words, the degree to which a monetary value is devalued over time. For this task each one of the 27 items is classified according to its k rank.

A similar procedure was used to assess probabilistic discounting. In this case the delay D is replaced by the odds against winning, Θ = (1—*p*)/*p*, as reported in the Eq. (2) which shows exaggerated declining subjective values of probabilistic outcomes^73^:

2 $$ V \, = \, A \, / \, \left( {1 \, + \, h\Theta } \right). $$

In both the MCQ, and the PDQ each one of the 30 items is classified according to its h rank. Thus, we obtained an individual k value—for which the higher the k value, the more steeply the individual discounts rewards delayed in time—and an individual h value—for which the lower the h value the higher is the value attributed to the probabilistic, rather than to the certain outcome.

### NBF analysis

Taking into account the interpersonal variation (i.e. metabolism level) and intra-training session (i.e. hydration) the data concerning the two parameters used to determine the effects of training, temperature and skin conductance were analysed as follows:

Baseline amplitude was calculated over + 30 s and + 60 s in 2 min rest data recording to provide an indication of the ongoing activity present when in testing position at rest.

Latency was defined as the instant lasting for at least 50 ms when its parameters amplitude was greater (temperature) or smaller (skin conductance) than the mean of its baseline value plus 2 standard deviations^74^.

Efficacy was defined as the greatest spread between the baseline value and peak skin conductance, lowest temperature and EEG values reached during the test session. All these values were calculated in rest, stress, and recovery conditions in accordance with the provided protocols.

### Statistical analysis

The Shapiro–Wilk test was conducted to verify that all data were normally distributed. An unpaired t-test was performed to assess potential differences at the baseline among variables. A two-way mixed analysis of variance (ANOVA) with a within-condition (Time, pre and post) and between-condition (Condition, NBF-G and CTRL-G) factor was performed to assess potential interactions (Time × Condition) for each dependent variable for both Probabilistic and Temporal tasks. In case of significant differences in each of the variables at the baseline, an analysis of covariance (ANCOVA) was implemented to adjust for baseline conditions. Post-hoc analyses to compare pairs of means were conducted using the Bonferroni adjustment. As a measure of effect size for ANOVA, partial eta squared (pη^2^) was reported. The thresholds for small, moderate, and large effects were defined as 0.01, 0.06, and 0.14, respectively^55^.

A t-test for independent sample was then conducted to compare questionnaires’ scores between experimental and the control conditions.

All the conventional statistical analyses were performed using the IBM SPSS® Statistics software (v. 21, New York, U.S.A.). Data are shown as mean ± SD without covariate adjustments. An α value of 0.05 was set as the criterion level of significance.
